# Diabetic myonecrosis in a patient with hepatic cirrhosis: a case report and review of the literature

**DOI:** 10.1186/1752-1947-3-9305

**Published:** 2009-11-30

**Authors:** Leonardo G Mancillas-Adame, Jose G González-González, Joel O Jáquez-Quintana, Myrna A Cardoza-Torres, Alberto de la Fuente García

**Affiliations:** 1 Endocrinology Division, Dr. Jose Eleuterio Gonzalez University Hospital, Universidad Autonoma de Nuevo Leon, Monterrey, México; 2 Internal Medicine Division, Dr. Jose Eleuterio Gonzalez University Hospital, Universidad Autonoma de Nuevo Leon, Monterrey, México

## Abstract

**Introduction:**

Diabetic myonecrosis was first reported by Angervall and Stener in 1965. In its classical clinical expression, it affects type 1 diabetes mellitus patients with long-standing poor metabolic control and advanced chronic microvascular complications. A sudden-onset of severe pain in the region of the involved muscle, usually the quadriceps, is the typical clinical manifestation. Magnetic resonance imaging (MRI) confirms the clinical diagnosis; in some cases of diagnostic uncertainty, a muscle biopsy may be required.

**Case Presentation:**

We present the case of a 38 year-old Hispanic male from Mexico, with alcohol-induced hepatic cirrhosis (Child-Pugh C/MELD 45) and type 2 diabetes mellitus admitted to the emergency room due to hepatic encephalopathy with intense pain and an increase in volume of the left thigh. MRI showed edema and inflammatory changes of the quadriceps muscle with a hyperintense signal on T2-weighted images; in addition, there was a subacute hematoma.

**Conclusion:**

To the best of our knowledge, this is the first case of diabetic myonecrosis associated with and complicated by advanced hepatic cirrhosis reported in the literature.

## Introduction

Diabetic myonecrosis was first reported by Angervall and Stener in 1965 [1]. In its classical clinical expression, it affects people with type 1 diabetes mellitus with long-standing poor metabolic control and advanced chronic microvascular complications. The typical clinical manifestation is the sudden onset of severe pain in the region of the involved muscle, usually the quadriceps. Magnetic resonance imaging (MRI) confirms the clinical diagnosis. In some cases of diagnostic uncertainty, a muscle biopsy may be required. Management of diabetic myonecrosis involves bed rest, adequate pain control and improvement of diabetes metabolic control. It has a poor prognosis and is a reflection of severe and generalized vasculopathy that can produce critical and fatal complications in the short-term [2].

## Case presentation

A 38-year-old Hispanic male from Mexico, with alcohol-induced hepatic cirrhosis (Child-Pugh C/MELD 45) and type 2 diabetes mellitus was admitted to the emergency room due to deteriorating encephalopathy. His diabetes had been diagnosed 10 years before. Metabolic control during this time was very poor because of alchoholism. One month before being admitted to the emergency room, he experienced sudden onset of increasing localized pain in the left thigh while resting. He had no fever or chills. Relatives said he did not experience trauma, skin lesions and insect bites. Analgesics only mildly soothed the pain. In the following weeks, he was unable to walk. Common oral analgesics no longer delivered relief from moderate to severe pain. He remained at rest until three days before admission when the encephalopathy worsened. During physical examination, he had no fever. His blood pressure was at 110/70 mmHg, his pulse rate was at 112. His respiratory rate was 24 per minute. He was conscious and oriented with grade II encephalopathy. Upon examining the left thigh, a 10 × 10 cm mass was located in the anterior and external part of the middle portion of the thigh. This lesion was not well-defined. It was tender and associated with tense skin with erythema, ecchymoses and an increase in temperature. He The patient experienced increased pain in the thigh when moving the limb. There was no peripheral edema. Ollow's sign and Homman's sign were absent. On neurological examination, vibratory and pain perception were bilaterally decreased.

**Figure 1:**
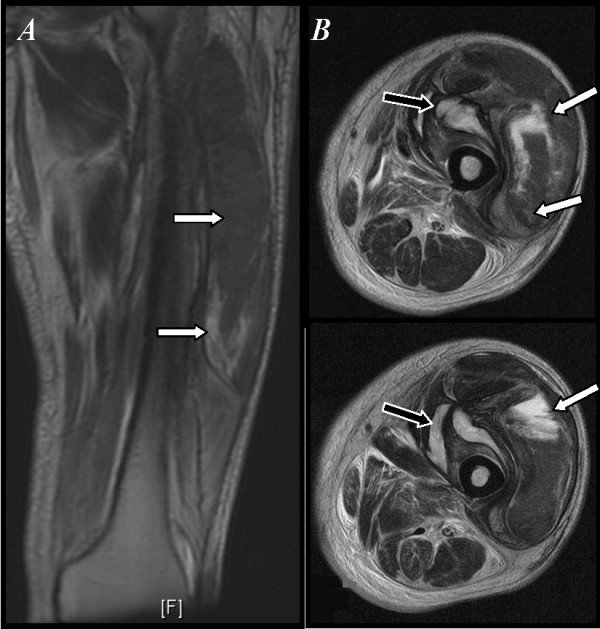
**Magnetic resonance imaging of the left thigh**. A) Coronal slice on T-2 sequence: hyperintense image of the quadriceps muscle in relation to inflammatory changes and necrosis (arrows). B) Axial slice on T-2 sequence: hyperintense image of the quadriceps muscle (white arrow), there is a fluid collection lateral to the vascular bundle (black arrow).

Blood testing (see table 1) showed a macrocytic, normochromic anemia with neutrophilic leucocytosis, thrombocytopenia and prolongation of coagulation times. A non-fasting plasma glucose value was elevated. Blood cultures and antiphospholipid antibodies were negative. X-ray imaging of the left thigh showed only soft tissue edema; a Doppler ultrasound ruled out deep vein thrombosis and an MRI showed edema and inflammatory changes of the quadriceps muscle with a hyperintense signal on T2-weighted images. There was also a subacute hematoma (Figure 1).

**Table 1:** Laboratory values on admission and some evolution landmarks

**Value**	**on admission**	**week 1**	**week 2**	**reference value**
Hematocrit (%)	22.8	18	25	37.7-53.7
Hemoglobin (g/dl)	7.9	6.36	8.79	12.2-18.1
Mean corpuscular volume (μm^3^)	101	104	98.4	80-97
Leucocytes (k/mm^3^)	17	9.290	7.270	4 - 11
Differential count (mm^3^)				
Neutrophils (k/mm^3^)	13.300	6.690	5.160	2.0-6.9
Lymphocytes (k/mm3)	1.440	1.030	0.977	0.6-3.4
Platelets (mm^3^)	46,000	41,100	11,400	140,000 - 425,000
Prothrombin time (s)	40.9	25	47	10.7-14.3
Partial thromboplastin time (s)	100	43	47	22.0-38.0
INR	5.4	3	4.8	
Erythrocyte Sedimentation rate (mm/hr)	16			< 10
Glucose (mg/dl)	145	85	102	70-100
Creatinine (mg/dl)	2.8	3.04	2	0.6-1.4
Proteins (gr/dl)	7.2		7	6.1-7.9
Albumin (g/dl)	1.8		1.7	3.5-4.8
Globulin (g/dl)	5.4		5.2	2.6-3.1
Aspartate aminotranspherase (U/Liter)	121		94	10.0-42
Alanine aminotranspherase (U/Liter)	72		63	10.0-42
Total bilirrubin (mg/dl)	11.14		11.8	0.2-1.0
Direct (mg/dl)	5		4.9	0.0-0.2
Alkaline Phosphatase (U/liter)	108		108	38-126
Creatine phosphokinase (U/liter)	138			22-262

Once diabetic myonecrosis and subacute hematoma were diagnosed, conservative management was initiated. Treatment included complete bed rest, parenteral analgesics and antibiotics to prevent spontaneous bacterial peritonitis. Therapy for hepatic encephalopathy, decompensated liver cirrhosis, and diabetes was also provided. There was a rapid improvement in pain control and resolution of the edema and the area of cellulitis. Five days later, however, there was increased pain in the erythematous area of the thigh. He also experienced low grade fever. An ultrasound guided muscle biopsy revealed only necrosis. Gram stain and cultures for anaerobic and aerobic bacteria and fungi were negative. During the procedure, partial evacuation of the subacute hematoma was carried out. After a slow and progressive improvement of the mass and edema, he was discharged from the hospital. Three weeks later, he came back to the emergency room with grade IV encephalopathy. Examination of the limbs did not reveal any abnormalities. Progressive deterioration of hepatic function led to severe liver insufficiency and death four days after admission.

## Discussion

Myonecrosis related to diabetes mellitus is an uncommon disorder. To date, only about 100 cases have been published. Severe diabetic microangiopathy has been proposed as the underlying mechanism that leads to spontaneous, non-gangrenous and focalized muscle infarction. There has also been some evidence that abnormalities in fibrinolysis and coagulation, embolic phenomenon and vasculitis can contribute in a minority of cases. This case is notable because of the association of diabetic myonecrosis with liver cirrhosis. This is the first reported case where both disorders were associated. Prolonged clotting time secondary to severe chronic liver damage almost surely contributed to formation of the hematoma, a complication that has not been previously observed in a patient with diabetic myonecrosis. This complication made the risk of infection likely. Evacuation of the hematoma in the presence of low grade fever during hospitalization was performed based on a high risk of infection. Despite improvement of this problem and the encephalopathy, it is possible that some kind of occult or asymptomatic infection contributed to the rapid deterioration of hepatic function, leading to irreversible and fatal hepatic insufficiency. Diabetic myonecrosis is a little known complication of diabetes, which is associated with a poor prognosis, requiring a high degree of suspicion.

Diabetic myonecrosis is a sporadic microvascular complication favored by poor metabolic control. It is related to other complications of diabetes mellitus, such as nephropathy (71%), retinopathy (56%) and neuropathy (54%) [1-3]. Long-term diabetic vasculopathy is essential for developing diabetic myonecrosis [4]. Classically, it has been reported in women with long-standing diabetes (15 years from diagnosis); type 1 diabetes is present in most cases (71%) [1,2,5]. In type 2 diabetes cases, it characteristically affects elderly patients.

Although not well defined, atheroembolism of small vessels has also been proposed as the responsible mechanism. It is likely that preference for the thigh muscles is due to the presence of multiple collateral vessels. Another proposed theory is the participation of the coagulation cascade with relation to the coagulation-fibrinolysis system [2,6].

Sudden onset of intense pain of the involved muscle of the patient is the usual initial manifestation. There is clear predilection for the thigh muscles; the disease rarely involves the muscles bilaterally. In most cases, a diffuse, rapidly progressive pain radiates to the low back, the knee and foot, making daily activities impossible. Fever has been reported in only 3% of subjects [1-7]. An increase in volume of the thigh is the first clinical sign. At the beginning it is not well delineated, but later it is localized to the implicated muscle. It may be associated with joint effusion. A palpable mass occurs in 40% of patients at presentation. Nerve compression is rare and motility restriction of the limb is a result of intense pain [5,7]. The *quadriceps cruralis *is the most commonly involved muscle (87%) and the *vastus lateralis *and *vastus medialis *are implicated in 24% and 22%, respectively. The abductors, hip flexors and muscles of the upper limb are rarely affected. Bilateral disease has been reported in about 10% of cases [6].

Lab testing is unhelpful. Creatine phosphokinase levels are normal or slightly elevated in 52%; leukocytosis occurs in 14% of cases. AST, ALT, LDH levels and the erythrocyte sedimentation rate are normal most times [8,5]. Deep vein thrombosis is the main differential diagnosis. Second place is occupied by entities that need to be ruled out, such as hematomas, pseudothrombophlebitis, acute arterial occlusion, abscesses, cellulitis, fascitis, bursitis and diabetic pyomyositis. It is sometimes difficult to distinguish diabetic myonecrosis from lumbosacral radiculoplexopathy, diabetic osteomielitis, muscle rupture, tumors, polymyositis or proliferative myositis.

A high degree of suspicion is required. With physical examination, it is difficult to identify the disease. Studies such as x-ray imaging, Doppler ultrasound, contrasted CT scan, angiography and MRI usually rules out other etiologies. MRI usually defines the diagnosis of diabetic myonecrosis. Magnetic resonance imaging shows a hyperintense signal in the T2 sequence of the involved muscle. Perimuscular or subcutaneous tissue edema are common. A hyperintense signal in the T1 sequence is sometimes observed, probably due to hemorrhage secondary to necrosis [9,10]. In very rare cases, a biopsy is required to confirm the diagnosis, mostly because of a lack of response to standard therapy [8,6,10].

Biopsy is the gold standard for diagnosis. But before undergoing it, there must be a clear indication, due to the risk of complications. A fine-needle percutaneous biopsy is preferred to open biopsy because of the risk of infection or hematoma [10,3]. Review of the biopsy shows areas of hemorrhagic necrosis and myositis with muscle fibers in different stages of degeneration-regeneration. The walls of small vessels are hyalinized with partial or total occlusion of the vessel lumen. In medium-sized vessels, atherosclerosis is observed [4,2,6,3]. Cultures for bacteria, fungi and acid-fast bacilli should be performed, but are negative unless complicated myonecrosis has taken place.

Control of pain and inflammation are the mainstay of therapy. Absolute bed rest and the use of non-steroidal anti-inflammatory drugs or narcotics can have a slow but progressive improvement. The use of concurrent anti-platelet and anti-inflammatory drugs may accelerate total recovery time from eight to five weeks [4,3]. Better metabolic control of the diabetic state is recommended; although there is no evidence that this will prevent morbidity or mortality. A hypercoagulable state should be considered and anticoagulation started if confirmed [6,3]. Surgical treatment is recommended for decompression in case of arterial compromise [8].

Most of the time, the short-term prognosis is excellent. Less than eight weeks are needed for pain and inflammation to disappear. The main underlying difficulty is advanced generalized microvasculopathy leading to life-threatening complications in a short period [2,11]. Up to 50% of cases may have a recurrence, most of them in a previously affected muscle [2,6]. Almost half of these recurrent events occur in a period of two months [12].

## Conclusion

This is the first case of diabetic myonecrosis associated with and complicated by advanced hepatic cirrhosis reported in the literature.

## Competing interests

The authors declare that they have no competing interests.

## Authors' contributions

LM & GG prepared the manuscript. JJ, AF and MC participated in literature review and case preparation. LM and JJ made the diagnostic procedures for myonecrosis and conceived the idea of a case report publication.
